# metaBEETL: high-throughput analysis of heterogeneous microbial populations from shotgun DNA sequences

**DOI:** 10.1186/1471-2105-14-S5-S2

**Published:** 2013-04-10

**Authors:** Christina Ander, Ole B Schulz-Trieglaff, Jens Stoye, Anthony J Cox

**Affiliations:** 1 Genome Informatics, Faculty of Technology and CeBiTec, Bielefeld University, Bielefeld, Germany; 2 Computational Biology Group, Illumina Cambridge Ltd., Chesterford Research Park, Little Chesterford, Essex CB10 1XL, United Kingdom

## Abstract

Environmental shotgun sequencing (ESS) has potential to give greater insight into microbial communities than targeted sequencing of 16S regions, but requires much higher sequence coverage. The advent of next-generation sequencing has made it feasible for the Human Microbiome Project and other initiatives to generate ESS data on a large scale, but computationally efficient methods for analysing such data sets are needed.

Here we present metaBEETL, a fast taxonomic classifier for environmental shotgun sequences. It uses a Burrows-Wheeler Transform (BWT) index of the sequencing reads and an indexed database of microbial reference sequences. Unlike other BWT-based tools, our method has no upper limit on the number or the total size of the reference sequences in its database. By capturing sequence relationships between strains, our reference index also allows us to classify reads which are not unique to an individual strain but are nevertheless specific to some higher phylogenetic order.

Tested on datasets with known taxonomic composition, metaBEETL gave results that are competitive with existing similarity-based tools: due to normalization steps which other classifiers lack, the taxonomic profile computed by metaBEETL closely matched the true environmental profile. At the same time, its moderate running time and low memory footprint allow metaBEETL to scale well to large data sets.

Code to construct the BWT indexed database and for the taxonomic classification is part of the BEETL library, available as a github repository at git@github.com:BEETL/BEETL.git.

## Background

Isolating and culturing individual members of a microbial population gives little insight into their relative abundances in the community and excludes entirely the majority of microorganisms that are difficult or impossible to culture. Metagenomic studies therefore seek to describe a microbial ecosystem in its full complexity by sampling its DNA directly. Early work targeted 16S and other ribosomal RNA genes for sequencing, since they are widely present across species but with a sequence diversity sufficient to serve as a marker for the presence of a given species. This is still a popular experimental design: extensive databases of species-specific 16S sequences are available [1] and mature tools exist for the analysis of such data [2]. However primer design issues, copy-number variation of the 16S gene and chimera formation can all confound the generation of accurate taxonomic profiles from 16S data [3,4]. Most seriously, 16S sequencing is blind to any genetic variation that lies outside the 16S region.

Such considerations motivate an interest in *environmental shotgun sequencing *(ESS), where each read can potentially come from anywhere in the genomes of the sampled microbes. Early ESS studies used *de novo *assembly to characterize the bacterial population in poorly-understood environments such as the Sargasso Sea [5] but large-scale initiatives such as the Human Microbiome Project [6], facilitated by a precipitous drop in the cost of DNA sequencing, have since made it reasonable to assume, in many cases of interest, that the sample we wish to analyze is a mixture of well-characterized strains for which we have reasonably complete genome sequences.

Nevertheless, the analysis of such data sets remains challenging. The most widely-used comparison-based taxonomic classifiers [7,8] rely on a version of BLAST [9] to align reads to a set of references, which is often a prohibitive computational overhead for the large ESS data sets generated by 'next-generation sequencing' (NGS) platforms. A similar bottleneck arises when NGS data sets are aligned to a reference genome sequence and has been met by a new generation of alignment tools, many of which achieve their efficiency by converting the reference genome to an index data structure based on the Burrows-Wheeler Transform (BWT). The tool Genometa [10] leverages these advances by first using either Bowtie [11] or BWA [12] to align ESS reads to a set of bacterial genome sequences and then post-processing the resulting alignments into taxonomic assignments.

In this paper we present metaBEETL, an algorithm for the taxonomic classification of ESS data that uses BWT indexing in a different way. First, we build an augmented index of the bacterial genomes that enables us to classify a read even if it cannot be unambiguously matched to an individual strain, by simply moving up in the Tree of Life until we arrive at a taxonomic level where the match is unambiguous. Second, we also index the reads themselves. This enables us to exploit redundancy present in the reads since any *k*-mers occurring in multiple reads are only compared once to the reference genome, so we see a sublinear gain in processing time as the number of reads we match increases.

To test the accuracy of metaBEETL we simulated an artificial metagenome and classified its reads using metaBEETL, comparing the results against classifications from CARMA3 [8], MEGAN [7] and Genometa [10].

## Methods

The Burrows-Wheeler Transform (BWT) [13,14] permutes the characters of a piece of text into a new string that not only tends to be more *compressible *than the original text but is also *reversible*, in that the original text can be deduced solely from its BWT. The combination of these two properties is remarkable and has made the BWT a core concept in data compression - in particular, it is at the heart of *compressed index *data structures such as the FM-index [15] which store text in a compressed form that also permits rapid searching for query strings within the data.

Each symbol in a BWT has an *associated suffix *in the string it was created from, such that the *i*-th character of the BWT is associated with the suffix of the string that is *i*-th smallest, if all its suffixes are placed in lexicographic order. The symbols whose associated suffixes start with some string *Q *form a single contiguous substring of the BWT that we call the *Q*-*interval *and express as a pair of coordinates [*b_Q_*, *e_Q_*] denoting the first and last character of the substring.

### Reference database

To index a collection of genomes we must first generalize the concept of the BWT from a single string to a collection of *n *texts. A straightforward way to do this is to imagine each member of the collection is terminated by a distinct member of a set of *special characters *that satisfy $_1 _*<*... *<*$*_n _*and are lexicographically less than all symbols of the 'regular' alphabet that the rest of the text is drawn from. We build such a generalized BWT for the collection *G *comprising a set of microbial reference genome sequences {*g*_1_, ... *g_m_*} together with their reverse complements {$g_{1}^{r}$, ..., $g_{m}^{r}$}. This is a 'one-time' procedure that only needs to be repeated when genomes are added to (or removed from) the collection so our approach prioritises simplicity over efficiency: first we build the suffix arrays for all members of *G *(which can be done in parallel), then we merge them by reading the suffix arrays element-by-element from disk into a Fibonacci queue. Using copies of the sequences held in RAM, we determine the relative ordering between suffixes from different members of the collection. This enables us to build not only the generalized BWT but also arrays *A *and *C *such that the suffix at position *A*[*i*] of member *C*[*i*] of *G *is the *i*-th smallest suffix in the collection. Together, *A *and *C *form a *generalized suffix array *of *G*.

The elements of *C *are used as keys into an array *T *of 8-vectors such that *T*[*i*] = {*superkingdom, phylum, class, order, family, genus, species, strain*} describes the classification of the *i*-th member of *G *according to the NCBI taxonomy [16], each member of the 8-vector being an integer that in turn points to an entry in an array of names for the relevant taxa.

### Taxonomic classification

BWT-based aligners such as Bowtie and BWA facilitate rapid matching of a set of sequences to a reference genome by converting the genome to a compressed index and using error-tolerant modifications of the basic 'backward search' strategy to check for matches to individual query sequences. Each search requires essentially random access to the index files, which must therefore be held in RAM. In [17] it was shown that all queries can be searched for simultaneously within an index by building a separate index of the query sequences themselves then making a series of sequential passes through the two indexes. Accessing the indexes in a sequential way is cache-efficient if one or both of the indexes do fit in RAM, but importantly also makes it feasible to compare them while they are both held on disk, thus preventing available RAM from constraining the sizes of the indexes that can be compared. Moreover, indexing the query sequences exploits redundancy within them since each distinct *k*-mer is compared with the reference index exactly once, even if it has multiple occurrences among the queries. Efficient algorithms suitable for indexing the large numbers of short sequences in a typical NGS data set are given in [18].

The comparison of a set of reads *R *against a collection of genomes *G *makes at most *n *passes through BWT(*R*) and BWT(*G*), where *n *is the length of the longest read in *R *(our implementation assumes all reads are the same length, although this is not a prerequisite of the method). At stage *k*, the *Q*-intervals of all *k*-mers *Q *that are present in either or both of BWT(*R*) and BWT(*G*) are considered in lexicographic order. During this traversal, a lexicographically ordered list of the *Q*-intervals of all (*k *+ 1)-mers in *R *and *G *is computed and stored in files ready for the next iteration, as described in [17, Figure 2].

For each *k*-mer *Q *that is present in both *R *and *G*, we extract from *C *the subarray *C*[*b_Q_*], *C*[*b_Q _*+ 1], ... , *C*[*e_Q_*] whose elements encode the provenance of the symbols in the *Q*-interval [*b_Q_*, *e_Q_*] of BWT(*G*). The *k*-mer *Q *is classified at the highest taxonomic level *l *for which *T*[*C*[*b_Q_*]][*l*] = *T *[*C*[*b_Q _*+ 1]][*l*] = ... = *T*[*C*[*e_Q_*]][*l*]. Turning to BWT(*R*), the size $e_{Q}^{\prime }\;-\;\;b_{Q}^{\prime }+1$ of the *Q*-interval [$b_{Q}^{\prime }$,$e_{Q}^{\prime }$] gives the number of occurrences of *Q *in the reads.

At the end of stage *k*, therefore, we have computed the abundance and taxonomic classification of all *k*-mers that are present in the reads. To convert these individual data points into a taxonomic profile that reflects the correct microbial composition of the sample, two sources of potential bias are considered. First, copy number changes can lead to over- or underestimation of certain taxa in a taxonomic profile [19]. We reduce such effects by considering only *k*-mers that occur no more than once in any genome in *R*. Second, per-read statistics such as these must be normalized by genome size to obtain a statistic that reflects the relative abundance of microbial cells [20]. To achieve this, the occurrences of all *k*-mers specific to a given taxon are aggregated and then divided by the mean lengths of the genomes within that taxon.

The optimal *k *for a given experiment is determined empirically and depends on the accuracy and length of its reads: the greater specificity of longer *k*-mers is weighed against the fact that sequencing errors and genomic variations cause fewer reads to be classified as *k *becomes close to the read length.

## Results

### Reference database

We downloaded the set of all NCBI RefSeq microbial sequences [21] and the associated NCBI taxonomy [16] on October 2nd 2012. This comprised 2097 genomes from bacteria, viruses and archaea, from which plasmid sequences were excluded to reduce the possibility of wrong taxonomic profiles through bacterial conjugation and copy number variation of plasmids in different microbes. The BWT and generalized suffix array of the remaining 2020 sequences and their reverse complements were generated as described in Methods.

### Accuracy test on a simulated metagenome

We simulated a metagenome containing equal proportions of microbes from fifteen organisms whose genomes are present in the NCBI Nucleotide database (Table 1), having genome sizes ranging from 0.2Mbp to 11Mbp with an average of 3.3Mbp. MetaSim [20] was used to simulate 100000 Illumina read pairs of length 80bp. The simulated dataset is small but its size was chosen to allow the BLASTX alignments needed by MEGAN and CARMA3 to finish in reasonable time on the hardware available to us.

**Table 1:** Composition of simulated metagenomic dataset having an even distribution of microbes.

Name	Taxonomic id	Size	Fraction in simulation	Read count
Blattabacterium sp. str. BPLAN	600809	0.64 Mb	6.67%	2372

Borrelia hermsii DAH chromosome	314723	0.92 Mb	6.67%	3616

Candidatus Blochmannia pen. str. BPEN	291272	0.79 Mb	6.67%	3122

Candidatus Sulcia muelleri DMIN	641892	0.24 Mb	6.67%	3122

Candidatus Zinderia insecticola CARI	871271	0.21 Mb	6.67%	816

Catenulispora acidiphila DSM 44928	479433	10.47 Mb	6.67%	41950

Chloroflexus aggregans DSM 9485	326427	4.68 Mb	6.67%	18684

Clostridium sp. BNL1100	755731	4.61 Mb	6.67%	18248

Deinococcus radiodurans R1	243230	3.06 Mb	6.67%	12066

Escherichia coli DH1	536056	4.63 Mb	6.67%	18400

Fluviicola taffensis DSM 16823	755732	4.63 Mb	6.67%	18258

Frankia sp. CcI3	106370	5.43 Mb	6.67%	21282

Geobacter bemidjiensis Bem	404380	4.61 Mb	6.67%	18344

Mycoplasma pneumoniae M129	272634	0.82 Mb	6.67%	3286

Yersinia enterocolitica subsp. e. 8081	150052	4.62 Mb	6.67%	18548

#### Comparison of computational costs

Aligning the reads to a set of reference sequences dominates the computational cost of MEGAN and CARMA3. Of the configurations tested in [8], aligning the reads to the NCBI NR database with BLASTX maximised the number of reads correctly classified by both programs, so we did the same with our data. These alignments were done on a cluster of 100 nodes, each node having at least 124GB memory available. The number of cores per node varied between 2 to 48, each having a clock speed of 2.0GHz.

Genometa and metaBEETL both ran on a single CPU Intel Xeon machine having eight 3.0GHz cores and 64Gb of shared RAM, to which we had sole access for our tests. metaBEETL needed only 200Mb of RAM but its index of reference genomes and its temporary files were stored on an attached solid-state hard drive to facililate the large amount of disk I/O that metaBEETL needs to do. Timings for the four methods are given in Table 4: the very different computational requirements of the BLAST-based and BWT-based tools make a like-for-like comparison difficult, but the advantage of the BWT-based methods is clear: metaBEETL finishes an order of magnitude more quickly on a single CPU than the BLAST-based methods do on a 100 node cluster.

Genometa, whose compute time is predominantly taken up by BWA alignments, is in turn an order of magnitude faster than metaBEETL, but our prototype implementation has considerable scope for optimization. At the moment, the reference BWT string is stored as ASCII, whereas a compressed format would greatly reduce the I/O that dominates metaBEETL's runtime. Moreover, it is likely that any given sample will only contain a small proportion of the 2020 genomes that are present in the database. Therefore, indexing the BWT string of the reference database should reduce I/O still further by allowing metaBEETL to jump directly to the relevant areas of the BWT instead of reading the entire string on every pass.

#### Comparison of correctly classified reads

CARMA3, MEGAN and Genometa were run with default parameters and metaBEETL was run with a *k*-mer length of 50. Table 2 shows reads correctly and incorrectly classified by the four tools at all taxonomic levels. The smaller number of reads classified by metaBEETL compared with CARMA and MEGAN3 is likely explained by metaBEETL's discarding of *k*-mers occurring multiple times in a single genome and by the fact that metaBEETL's database is a subset of the NCBI NR database used by the other two tools. Genometa requires a curated database (only one reference per genus, for instance) and we thus had to use the database available for download from the Genometa webpage. We manually checked that all the genomes used in the simulated sample were contained in this database. Importantly, metaBEETL is the best of the four tools in correctly classifying reads at the species level and misclassifies the fewest reads at all taxonomic levels.

**Table 2:** Comparison of the correctly classified (true positive - TP) and not correct classified (false positive - FP) reads of the simulated metagenome between the classifiers, metaBEETL, CARMA3, MEGAN and Genometa.

Taxonomic Level	metaBEETL		CARMA3		MEGAN		Genometa	
	TP	FP	TP	FP	TP	FP	TP	FP
Superkingdom	129,290	0	161,162	153	178,712	5	118,340	0

Phylum	129,280	10	158,904	395	176,604	31	113,138	5,202

Class	129,279	11	157,545	395	175,718	42	113,138	5,202

Order	129,279	11	155,625	220	174,737	47	113,138	5,202

Family	129,278	11	151,684	363	171,227	103	113,125	5,208

Genus	129,262	28	132,251	513	151,292	649	109,884	8,435

Species	129,242	48	51,920	232	110,728	1,196	109,444	8,896

#### Comparison of taxonomic profiles

An obvious way to assess the performance of a metagenomic classifier is simply to count the number of correctly classified reads, but we have already observed that copy number changes and different genome sizes can prevent the relative read counts from correctly reflecting the relative abundances of the microbes they are sequenced from. For this reason we decided not to perform comparisons solely based on the number of classified reads but also based on the expected taxonomic profile. We used the Euclidean distance $\sqrt{\overset{n}{\underset{i=1}{\sum }}{\left(q_{i}-p_{i}\right)}^{2}}$ to compute the distance between computed and simulated taxonomic profile and the results can be found in Table 3. We can see that metaBEETL produces a taxonomic profile which is much closer to the simulated ground truth than the other classifiers. For Genometa, we could only generate the taxonomic profiles at the genus and species level, because Genometa does not produce higher level taxonomic classifications. The taxonomic profiles for the levels superkingdom to species can be found in the additional files 1,2, 3, 4,5, 6.

**Table 3:** Comparison of the simulated taxonomic profile of an artificial metagenome and the predicted profiles from metaBEETL, CARMA3, MEGAN and Genometa.

Taxonomic Level	metaBEETL	CARMA3	MEGAN	Genometa
Superkingdom	1	1	1	-

Phylum	7.47	22.44	22.89	-

Class	7.48	25.70	23.84	-

Order	9.45	24.26	24.23	-

Family	9.39	22.15	19.60	-

Genus	10.85	26.22	21.56	38.82

Species	10.59	19.02	22.44	38.16

We compared profiles using the Euclidean distance to the simulated profile. Results from Genometa were only available at level genus and species.

**Table 4:** Running time and memory requirements of the tested classifiers on the simulated data set.

	metaBEETL	CARMA3	MEGAN	Genometa
Memory	1 GB	13 GB	13 GB	3 GB

Time	46 m	18 h 35 m	14 h 58 m	2 min

CARMA3 and MEGAN were run on a compute cluster, using 100 nodes. metaBEETL was run on an SSD drive. Memory consumption was taken at peak memory usage for one thread. All times are taken as wall clock times. For CARMA3 and MEGAN the time for the longest running time of the 100 threads was taken, the average time for MEGAN was 12 h 15 m and for CARMA 12 h 30 m.

It can be seen that metaBEETL gives a taxonomic profile which is closer to the true taxonomic profile than the ones from CARMA3, MEGAN and Genometa. The difference in the classification originates from under- as well as overestimation of different taxa from CARMA3, MEGAN and Genometa. For CARMA3 and MEGAN the differences in classification cannot be explained by the difference in the comparison databases, because the NCBI NR database includes the reference sequences used by metaBEETL. On the other hand the difference in database may be the cause for the higher distance from the true profile to the one produced by Genometa. By using a curated database with a limited size, Genometa loses the advantage of having a broader spectra of references. Therefore even though Genometa was much faster in the analysis of the simulated metagenome, the resulting taxonomic profile shows the most differences to the simulated profile. We could compare those profiles only at the genus and species level, because Genometa does not provide higher taxonomic classifications. metaBEETL performed much faster than CARMA and MEGAN, while using less memory, which makes it possible to analyze large ESS data sets. That metaBEETL is nearer to the true taxonomic profile shows that the bias reduction through removing sequences which occur more than once in the genome and the normalization gives metaBEETL an advantage over other classifiers.

### Accuracy testing of metaBEETL on a modified database

A key challenge of metagenomic studies is that the majority of microbes cannot be grown as a single culture and so are likely to be absent from our database of reference genomes. To test metaBEETL's ability to classify reads from genomes that are missing from its database, we masked all microbial reference sequences in the database that share the same species as the microbes in our simulated metagenome. A correct classification at species or strain level clearly becomes impossible, but we would still like to see good concordance with the expected profile at higher taxonomic levels. We found metaBEETL produced taxonomic profiles with Euclidean distances that ranged between 28 and 47 of the expected profile, and the concordance improved with higher numbers of simulated reads.

### Evaluation on real data

We downloaded sample SRS013948 from the Human Microbiome project, which comprises 31,107,576 reads from the throat of a male participant. Some reads had been quality trimmed and were padded with Ns to achieve a uniform read length of 100bp across the dataset. Building a BWT of the padded reads using our approach in [18] took around 4 hours, which could be reduced further if a solid-state drive had been used instead of shared disk storage. RAM usage was negligible.

**Figure 1:**
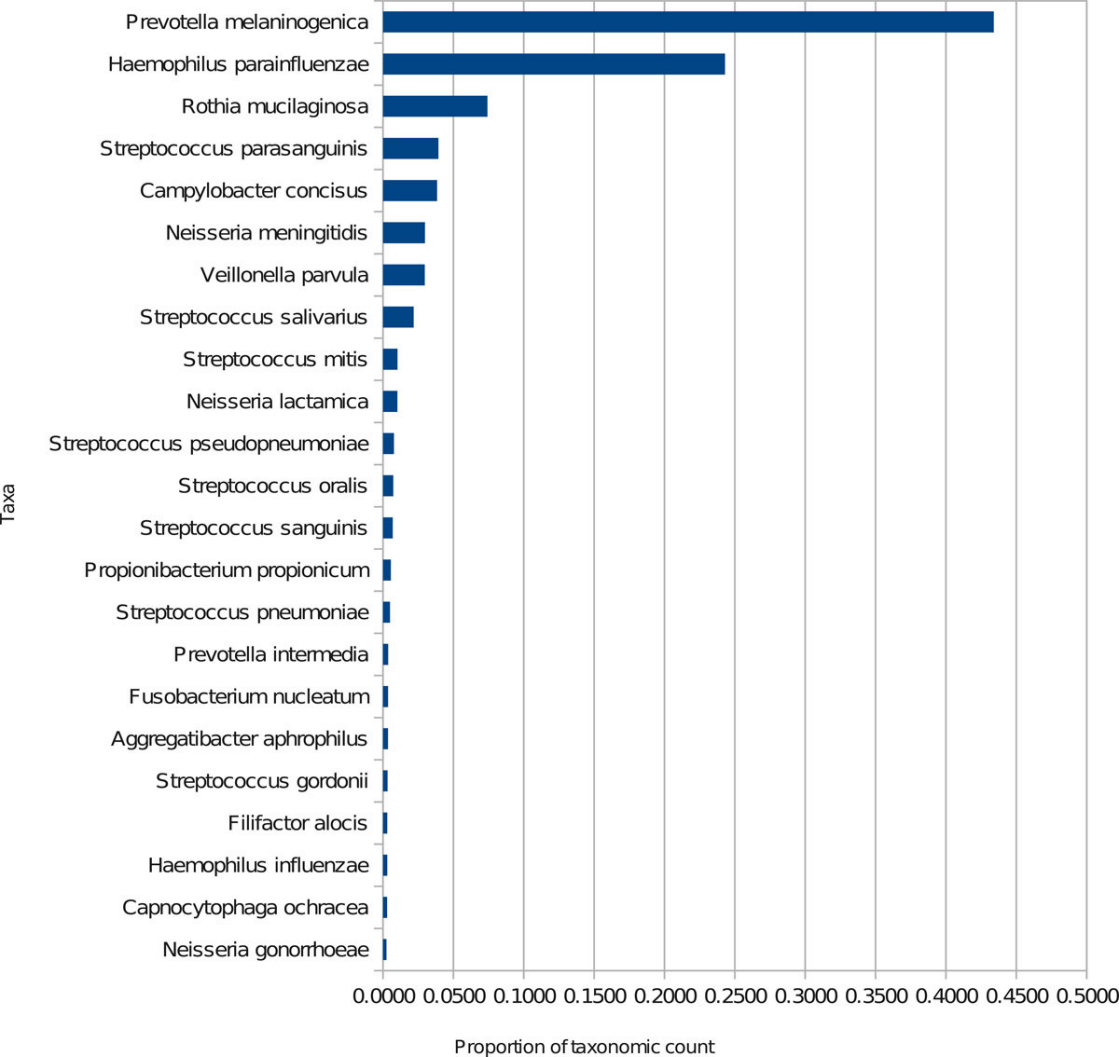
**Species-level classification computed by metaBEETL for sample SRS013948 from the Human Microbiome Project**.

Using a *k*-mer length of 75, metaBEETL took 42 hours to classify the reads, and the results are given in Figure 1. It can be seen that the taxonomic profile of this sample is highly uneven, with a few species dominating the environment. The most common assignment of the reads was to *Prevotella melaninogenica*, which is an oral opportunistic pathogen. Other highly abundant species are also known as pathogens occurring in lung, throat and mouth of humans. This could point to an infection of the human male from which the sample was taken.

## Conclusion

We presented metaBEETL, an algorithm for the taxonomic classification of sequencing reads from metagenomic shotgun experiments. metaBEETL uses indexed representations of both the input reads and the reference genomes they are compared against. We demonstrated on real and simulated data that its performance is competitive to BLAST-based metagenomic classifiers such as CARMA3 and MEGAN, while scaling better to the large data sets generated by next-generation sequencing technologies.

Like Genometa, metaBEETL relies on BWT-based text indexing, but there are fundamental differences in the two approaches. Genometa uses standard read mapping tools to perform its alignments, meaning its overall runtime is faster. However, the BWA and Bowtie aligners both have upper limits of around 3Gb on the total volume of reference sequence that they can index, which will become an issue as the number of available bacterial genome sequences increases. Moreover, this reliance also means its ability to handle ambiguous matches is limited: a strain from each species must be hand-chosen to be added to the index as an exemplar of that species. In contrast, the bespoke nature of our BWT index allows us to distinguish between different strains and to assign reads to a higher phylogenetic order when a strain-specific match is not possible.

In many ways, our current implementation does not fully exploit the information present in the indexes. Instead of relying on an empirically chosen *k*-mer size, a future version could aggregate information from multiple values of *k *to continue to extend only those sequences that are not yet long enough to be specific at the strain level. Moreover, *k*-mers that are specific to a given strain can be used to identify novel variants within that strain. Indeed, Simpson and Durbin [22,23] have shown that BWT indexes of reads facilitate fast and practical de novo assembly and a tool that combines reference-based classification with de novo assembly of unclassified reads is an intriguing future possibility.

The source code of metaBEETL is freely available from github as part of the BEETL software library.

## Competing interests

O.S-T. and A.J.C. are employees of Illumina Inc., a public company that develops and markets systems for genetic analysis, and receive shares as part of their compensation. Part of C.A.'s contribution was made while on a paid internship at Illumina's offices in Cambridge, UK.

## Authors' contributions

C.A. implemented the algorithm and performed computational experiments. O.S-T. performed experiments. A.J.C. designed and implemented the algorithm. C.A., O.S.-T., J. S. and A.J.C. wrote the manuscript. All authors have read and approved the manuscript.

## Supplementary Material

Additional File 1 **Phylum-level composition of simulated data compared with classifications produced by metaBEETL, CARMA3 and MEGAN**. Click here for file

Additional File 2 **Class-level composition of simulated data compared with classifications produced by metaBEETL, CARMA3 and MEGAN**. Click here for file

Additional File 3 **Order-level composition of simulated data compared with classifications produced by metaBEETL, CARMA3 and MEGAN**. Click here for file

Additional File 4 **Family-level composition of simulated data compared with classifications produced by metaBEETL, CARMA3 and MEGAN**. Click here for file

Additional File 5 **Genus-level composition of simulated data compared with classifications produced by metaBEETL, CARMA3 and MEGAN**. Click here for file

Additional File 6 **Species-level composition of simulated data compared with classifications produced by metaBEETL, CARMA3 and MEGAN**. Click here for file

## Declarations

The fees for the publication of this article were funded by Illumina Inc.

This article has been published as part of *BMC Bioinformatics *Volume 14 Supplement 5, 2013: Proceedings of the Third Annual RECOMB Satellite Workshop on Massively Parallel Sequencing (RECOMB-seq 2013). The full contents of the supplement are available online at http://www.biomedcentral.com/bmcbioinformatics/supplements/14/S5.
