# Supplementary material for: metaBEETL: high-throughput analysis of heterogeneous microbial populations from shotgun DNA sequences
Source: BMC Bioinformatics. 2013 Apr 10;14(Suppl 5):S2. doi: 10.1186/1471-2105-14-S5-S2 (PMC3622627; doi:10.1186/1471-2105-14-S5-S2)
Supplement: Additional File 3 — Order-level composition of simulated data compared with classifications produced by metaBEETL, CARMA3 and MEGAN. [file 1471-2105-14-S5-S2-S3.PDF]

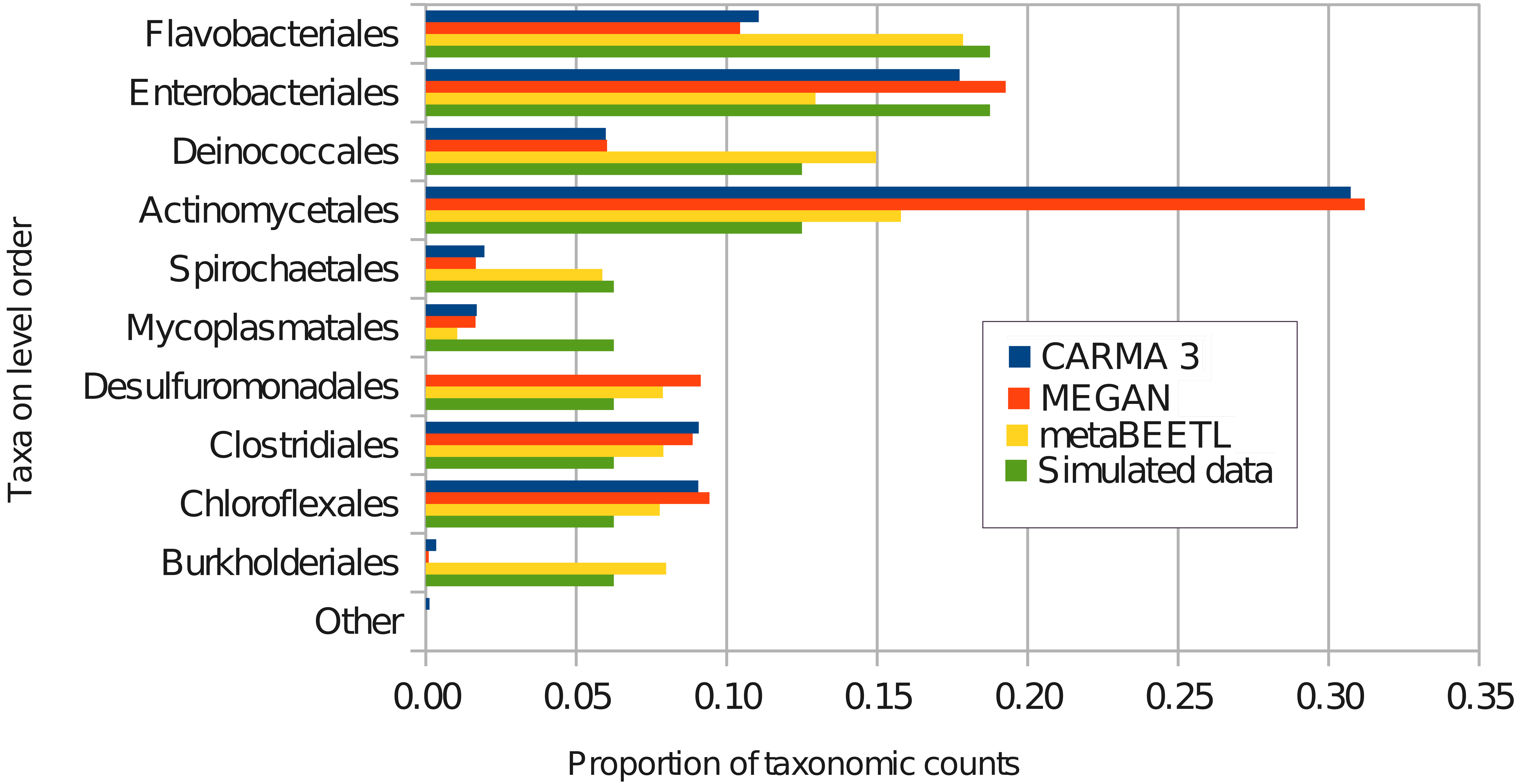
Flavobacteriales
Enterobacteriales
Deinococcales
Actinomycetales
Spirochaetales
Mycoplasmatales
Desulfuromonadales
Clostridiales
Chloroflexales
Burkholderiales
Other
Taxa on level order
0.00
0.05
0.10
0.15
0.20
0.25
0.30
0.35
Proportion of taxonomic counts
CARMA 3
MEGAN
metaBEETL
Simulated data
