# Supplementary material for: metaBEETL: high-throughput analysis of heterogeneous microbial populations from shotgun DNA sequences
Source: BMC Bioinformatics. 2013 Apr 10;14(Suppl 5):S2. doi: 10.1186/1471-2105-14-S5-S2 (PMC3622627; doi:10.1186/1471-2105-14-S5-S2)
Supplement: Additional File 5 — Genus-level composition of simulated data compared with classifications produced by metaBEETL, CARMA3 and MEGAN. [file 1471-2105-14-S5-S2-S5.PDF]

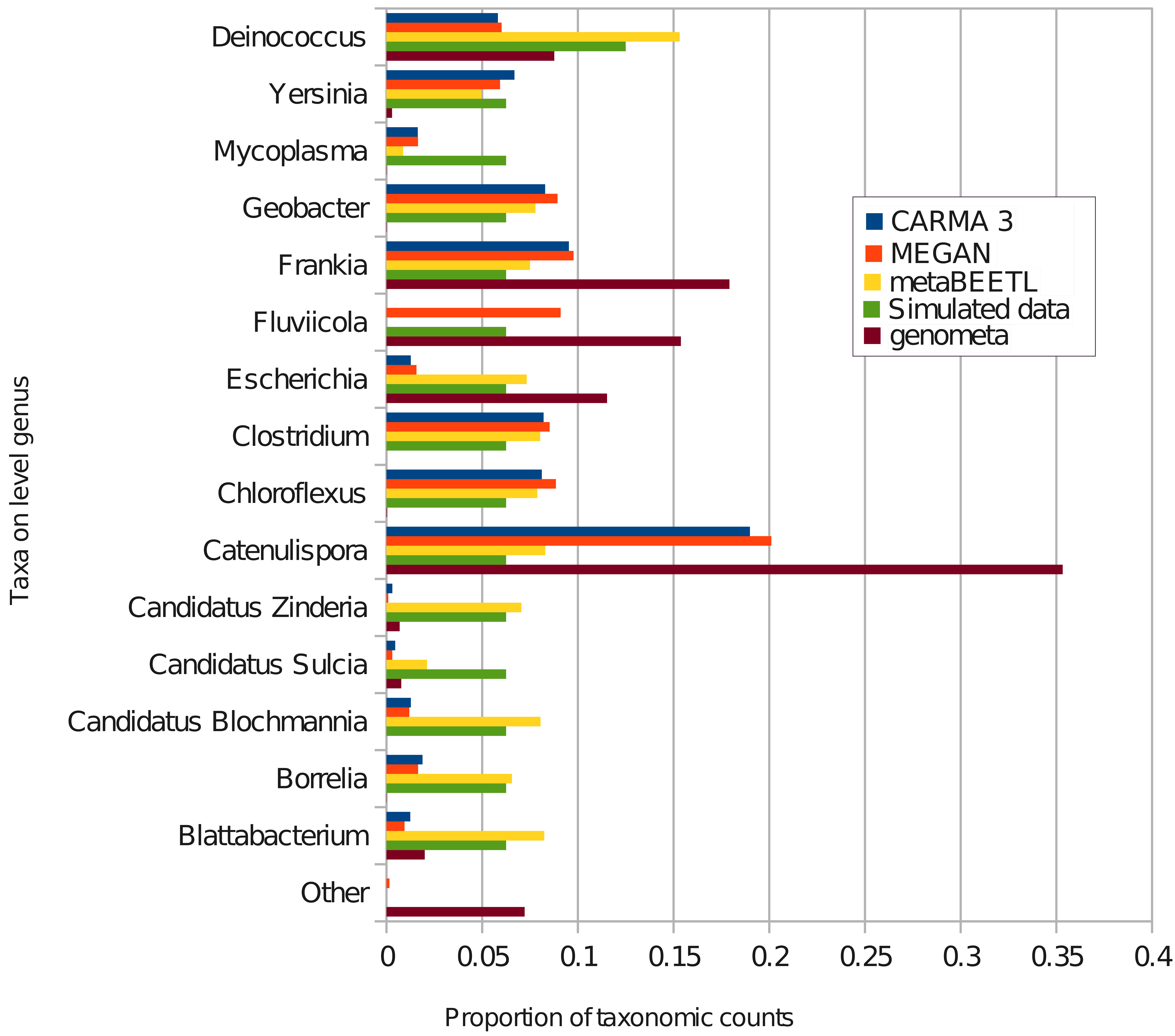
Deinococcus
Yersinia
Mycoplasma
Geobacter
Frankia
Fluviicola
Escherichia
Clostridium
Chloroflexus
Catenulispora
Candidatus Zinderia
Candidatus Sulcia
Candidatus Blochmannia
Borrelia
Blattabacterium
Other
Taxa on level genus
0
0.05
0.1
0.15
0.2
0.25
0.3
0.35
0.4
Proportion of taxonomic counts
CARMA 3
MEGAN
metaBEETL
Simulated data
genometa
