# Supplementary material for: metaBEETL: high-throughput analysis of heterogeneous microbial populations from shotgun DNA sequences
Source: BMC Bioinformatics. 2013 Apr 10;14(Suppl 5):S2. doi: 10.1186/1471-2105-14-S5-S2 (PMC3622627; doi:10.1186/1471-2105-14-S5-S2)
Supplement: Additional File 6 — Species-level composition of simulated data compared with classifications produced by metaBEETL, CARMA3 and MEGAN. [file 1471-2105-14-S5-S2-S6.PDF]

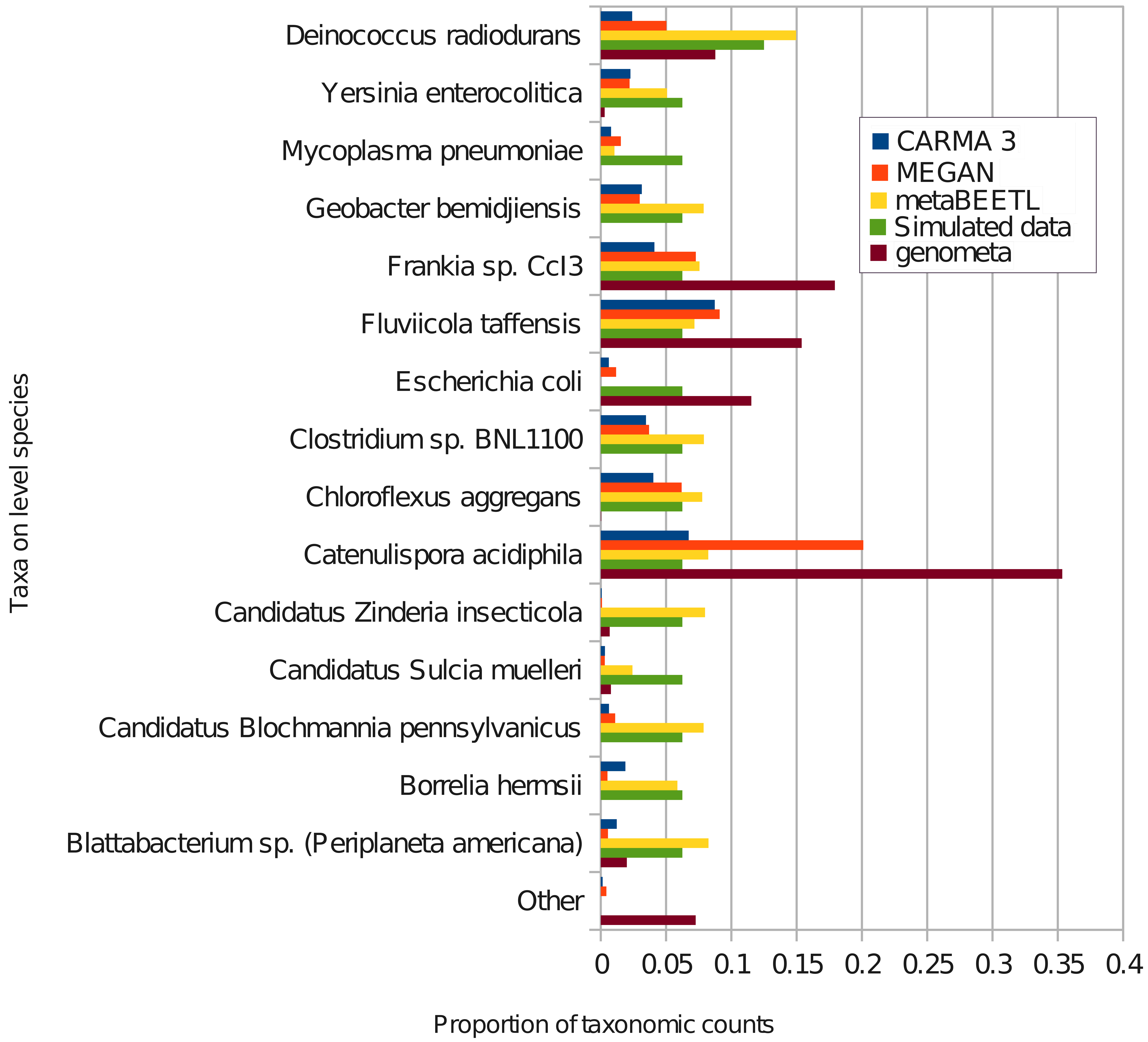

Deinococcus radiodurans
Yersinia enterocolitica
Mycoplasma pneumoniae
Geobacter bemidjiensis
Frankia sp. CcI3
Fluviicola taffensis
Escherichia coli
Clostridium sp. BNL1100
Chloroflexus aggregans
Catenulispora acidiphila
Candidatus Zinderia insecticola
Candidatus Sulcia muelleri
Candidatus Blochmannia pennsylvanicus
Borrelia hermsii
Blattabacterium sp. (Periplaneta americana)
Other
CARMA 3
MEGAN
metaBEETL
Simulated data
genometa
0
0.05
0.1
0.15
0.2
0.25
0.3
0.35
0.4
Taxa on level species
Proportion of taxonomic counts
